# Gene-Centric Characteristics of Genome-Wide Association Studies

**DOI:** 10.1371/journal.pone.0001262

**Published:** 2007-12-05

**Authors:** Changzheng Dong, Ziliang Qian, Peilin Jia, Ying Wang, Wei Huang, Yixue Li

**Affiliations:** 1 Bioinformatics Center, Key Lab of Systems Biology, Shanghai Institutes for Biological Sciences, Chinese Academy of Sciences, Shanghai, China; 2 Chinese National Human Genome Center at Shanghai, Shanghai, China; 3 Graduate School of the Chinese Academy of Sciences, Beijing, China; 4 Rui Jin Hospital, School of Medicine, Shanghai Jiaotong University, Shanghai, China; 5 Shanghai Center for Bioinformation Technology, Shanghai, China; National Institute of Neurological Disorders and Stroke, United States of America

## Abstract

**Background:**

The high-throughput genotyping chips have contributed greatly to genome-wide association (GWA) studies to identify novel disease susceptibility single nucleotide polymorphisms (SNPs). The high-density chips are designed using two different SNP selection approaches, the direct gene-centric approach, and the indirect quasi-random SNPs or linkage disequilibrium (LD)-based tagSNPs approaches. Although all these approaches can provide high genome coverage and ascertain variants in genes, it is not clear to which extent these approaches could capture the common genic variants. It is also important to characterize and compare the differences between these approaches.

**Methodology/Principal Findings:**

In our study, by using both the Phase II HapMap data and the disease variants extracted from OMIM, a gene-centric evaluation was first performed to evaluate the ability of the approaches in capturing the disease variants in Caucasian population. Then the distribution patterns of SNPs were also characterized in genic regions, evolutionarily conserved introns and nongenic regions, ontologies and pathways. The results show that, no mater which SNP selection approach is used, the current high-density SNP chips provide very high coverage in genic regions and can capture most of known common disease variants under HapMap frame. The results also show that the differences between the direct and the indirect approaches are relatively small. Both have similar SNP distribution patterns in these gene-centric characteristics.

**Conclusions/Significance:**

This study suggests that the indirect approaches not only have the advantage of high coverage but also are useful for studies focusing on various functional SNPs either in genes or in the conserved regions that the direct approach supports. The study and the annotation of characteristics will be helpful for designing and analyzing GWA studies that aim to identify genetic risk factors involved in common diseases, especially variants in genes and conserved regions.

## Introduction

Genome-wide association (GWA) studies using high-throughput single nucleotide polymorphism (SNP) chips have shown the power to identify novel disease susceptibility loci [1]–[3]. Two SNP selection approaches are proposed to design high-density chips: the direct approach and the indirect approach [4]–[7]. The direct gene-centric approach, which focuses on genetic variants in genic regions [4], [5] , can capture putative variants directly. The indirect approach using quasi-random SNPs or LD-based tagSNPs aims to capture most of the common variants in both genic and nongenic regions [6], [7]. It provides higher coverage of genome and explores genic variants as well as potential variants in regions outside known genes.

It is clear that both approaches can cover the genome densely either directly or through linkage disequilibrium (LD) [8], [9] and be successful in identifying disease variants in genes [1]–[3]. It is not clear, however, the extent to which these approaches can capture the common genic variants. Moreover, it is also important to characterize and compare differences among the approaches used in GWA studies. Nicolae et al. [10] investigated Affymetrix GeneChip Human Mapping 100K and found that SNPs in the set were undersampled from coding regions and oversampled from regions outside genes. Jorgenson and Witte [11] evaluated the coverage of both genic and nongenic SNPs, and estimated that random and tagSNP strategy for the indirect approaches could provide lower coverage of genic SNPs than nongenic SNPs. In this study, we perform a gene-centric evaluation to characterize the above approaches used in GWA studies.

Our evaluation is performed on three whole-genome commercial chips representing the above SNP-selection approaches: Illumina Human-1 Genotyping BeadChip (Human-1, gene-centric SNP panel) [12], Affymetrix GeneChip Human Mapping 500K Array Set (GeneChip 500K, quasi-random SNP panel) [13] and Illumina Human Hap550 (Hap550, LD-based tagSNP panel) [12]. By using both the empirical Phase II HapMap CEU data (Utah residents with ancestry from northern and western Europe) [14] and the disease variants extracted from OMIM [15], we evaluated the coverage of the approaches in genic regions and the ability to capture disease variants in Caucasian population. Since SNPs in diseases associated pathways and functionally important sequences (for example, genes and evolutionarily conserved region) are more attractive, we further compared the distribution patterns of SNPs with these characteristics. Our study reveals the common and different characteristics between the approaches used in GWA studies.

## Results

### Evaluating coverage and capturing disease variants in genic regions

**Figure 1:**
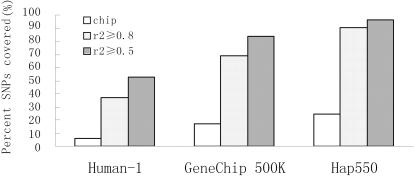
Coverage of three high-throughput SNP chips in genic regions. Three chips cover Phase II HapMap SNPs either directly (chip) or through linkage disequilibrium (r^2^≥0.8 and r^2^≥0.5).

**Figure 1** shows coverage of three high-throughput SNP chips in genic regions (MAF≥0.05, CEU). Although three chips cover about 6∼25% of Phase II HapMap SNPs directly (chip), the coverage increases quickly to 37∼96% when counting tagged SNPs (r^2^≥0.8 and r^2^≥0.5). Since Hap550 uses the tagSNPs selected from Phase I+II HapMap data set , it gets the coverage of near 91% (r^2^≥0.8) as expected. If r^2^ threshold set to 0.5, Human-1 and GeneChip 500K also gets the coverage of 53% and 84%, respectively. On average, Hap550 and GeneChip can get additional 3∼4 folds nonredundant LD SNPs; while Human-1 can get 5∼8 folds.

**Figure 2:**
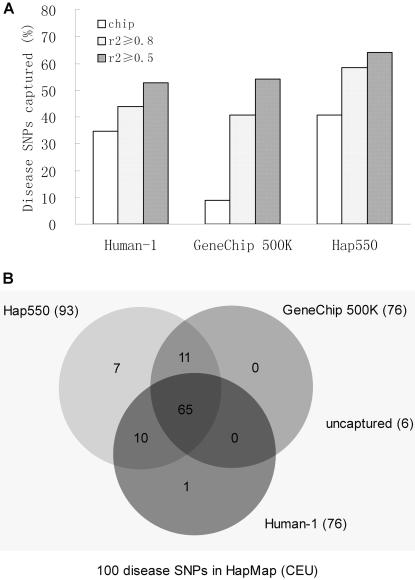
Disease variants in HapMap captured by three high-throughput chips. A. Shows the percentage of disease variants captured by three chips either directly (chip) or through linkage disequilibrium (r^2^≥0.8 and r^2^≥0.5). B. Displays the overlapped results captured by three chips (r^2^≥0.5).

There are totally 1338 nonredundant SNPs in OMIM that are defined as disease variants and associated with either diseases or phenotypes. Among these 1338 nonredundant SNPs, 159 of which can be mapped to the Phase II HapMap CEU data. We then evaluate the ability of SNP chips to capture 100 of 159 disease variants with MAF≥0.5. Human-1 and Hap550 can directly capture 48% and 62% of variants, respectively (**Figure 2A**), whereas GeneChip can only capture 11%. Via LD-tagging, all chips can capture more than 55% (r^2^≥0.8) and 75% (r^2^≥0.5) of SNPs. According to additional 59 disease variants with MAF<0.05, 75% of them are uncaptured (**Table S1**). **Figure 2B** shows the overlap of captured variants (r^2^≥0.5). There are 65 of SNPs captured by all SNP chips, whereas 6 of them are not captured by any chips. It shows that the indirect approaches have the same ability as the direct approach to capture most of common disease variants in genes in HapMap.

### Distribution patterns of SNPs in genic regions

**Figure 3:**
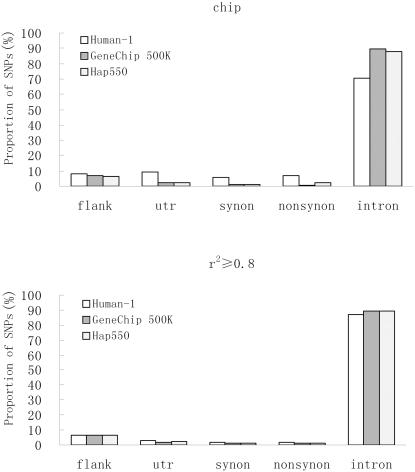
Distribution patterns of SNPs in genic regions. SNPs in genic regions are annotated with five functional classifications: flank (within 2 kb 5′ or 500 bp 3′ of a gene, originally named locus in dbSNP), utr (5′ and 3′ untranslated region), synonymous (synonymous coding SNP), nonsynonymous (nonsynonymous coding SNP) and intron (including splice-site SNPs).

SNPs are first classified into genic or nongenic regions with the annotation of dbSNP. In the analyses, less than 45% of SNPs in GeneChip 500K and Hap550 locate in genic regions, while 72% of Human-1 lie in genic regions (**Figure S1**). When counting tagged SNPs, the difference is reduced to near ten percent. SNPs in genic regions can further be classified into one of the five functional classifications: flank, utr, synonymous, nonsynonymous and intron. The distribution patterns of GeneChip 500K and Hap550 are very similar (**Figure 3**): similar high proportion of intron classification and similar distribution in other classifications. Almost 90% SNPs of GeneChip 500K and Hap550 locate in intron, meanwhile 7% SNPs lie in flank region, which makes it second-rich region. Each classification of utr, synonymous and nonsynonymous carries 1∼2 percent SNPs. As expected, Human-1 carries relative small proportion of SNPs in intron region (70%) and high proportions in other regions (5∼10%). It highlights the pertinent gene-centric design of the functional chip. Owing to the increasing proportion of intron region in Human-1, all three chips have the same distribution pattern when including tagged SNPs (r^2^≥0.8).

### Distribution patterns of SNPs in GO categories and KEGG pathways

**Figure 4:**
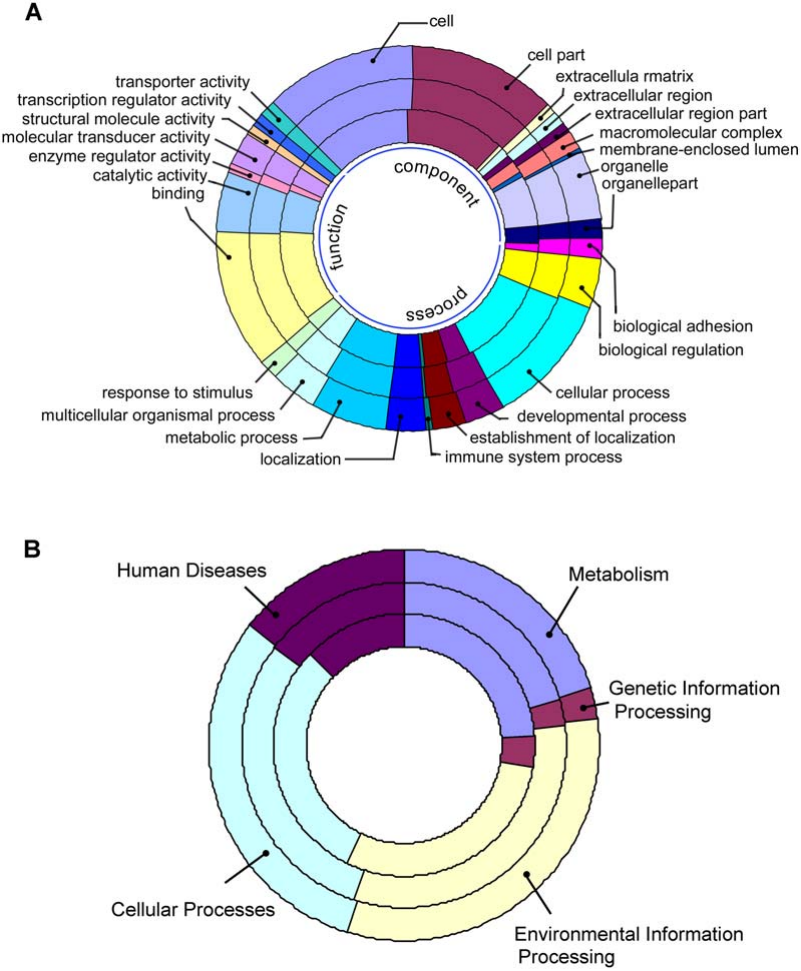
Distribution patterns of SNPs in ontologies and pathways. Outer rings: SNPs on Hap550. Middle rings: SNPs on GeneChip 500K. Inner rings: SNPs on Human-1. Each section represents the number of SNPs on the chips assigned to a given GO category or KEGG pathways. A. Distribution patterns in GO cellular component, molecular function and biological process categories. B. Distribution patterns in KEGG pathways.

To examine whether SNPs of Human-1, GeneChip 500K and Hap550 distribute in the same ontologies and pathways via genes, we mapped SNPs to gene ontology (GO) annotation [16] and Kyoto Encyclopedia of Genes and Genomes (KEGG) [17] pathways. The distribution of SNPs in all GO component, function and process categories (**Figure 4A**) and KEGG pathways (**Figure 4B**) remained largely the same between GeneChip 500K (middle rings) and Hap550 (outer rings), and slightly different with Human-1 (inner rings). We tested the hypothesis that equal percentages of SNPs on the chips distribute in KEGG human diseases pathways (**Table 1**). GeneChip 500K and Hap550 show no significant differences in all pathways except for pathogenic Escherichia coli infection pathway. However, significant differences are found between the direct and the indirect approaches. Human-1 exhibits enrichment in several pathways such as Huntington's disease and pancreatic cancer pathways, whereas GeneChip 500K and Hap550 enrich in Parkinson's disease, Dentatorubropallidoluysian atrophy (DRPLA) and non-small cell lung cancer pathways. Furthermore, the difference enlarges (**Table S2**) if LD-tagged SNPs are taken into consideration. More pathways exhibit significant difference between the direct and the indirect approaches.

**Table 1:** Distribution differences of SNPs (chip) in KEGG human diseases pathways.

Disease	Number of SNPs in pathways			Pairwise comparison p-value^1^		
	Human-1	500K	Hap550	Human-1: 500K	Human-1: Hap550	500K: Hap550
Alzheimer's disease	102	241	269	0.0412	**1.7E-05**	0.0039
Amyotrophic lateral sclerosis (ALS)	60	187	229	0.8215	0.3705	0.0972
Basal cell carcinoma	131	320	445	0.0456	0.014	0.6157
Cholera	122	332	494	0.3419	0.4852	0.6721
Chronic myeloid leukemia	247	595	869	0.0039	0.0041	0.8283
Colorectal cancer	306	852	1251	0.2512	0.3478	0.7086
Dentatorubropallidoluysian atrophy (DRPLA)	117	584	828	**7.1E-07**	**9.4E-07**	0.7463
Endometrial cancer	239	880	1353	0.0075	**2.5E-04**	0.1582
Epithelial cell signaling in Helicobacter pylori infection	214	511	716	0.0051	**9.7E-04**	0.6152
Glioma	248	698	1077	0.3688	0.9832	0.1811
Huntington's disease	112	197	227	**5.3E-06**	**2.1E-11**	0.0213
Maturity onset diabetes of the young	46	93	165	0.0266	0.2494	0.1122
Melanoma	239	740	1024	0.7226	0.8386	0.3951
Neurodegenerative Disorders	162	637	886	0.0029	0.0084	0.4862
Non-small cell lung cancer	218	986	1508	**1.1E-07**	**3.2E-10**	0.1731
Pancreatic cancer	255	579	766	**2.3E-04**	**5.4E-07**	0.1213
Parkinson's disease	69	354	516	**5.0E-05**	**2.2E-05**	0.8882
Pathogenic Escherichia coli infection	91	357	367	0.0259	0.5231	**5.8E-06**
Prion disease	55	125	201	0.0821	0.255	0.3452
Prostate cancer	273	739	1147	0.1415	0.6181	0.1344
Renal cell carcinoma	223	573	782	0.0485	0.0055	0.3185
Small cell lung cancer	425	1306	1901	0.738	0.6184	0.823
Thyroid Cancer	85	262	362	0.8575	0.8627	0.5938
Type I diabetes mellitus	117	316	498	0.3177	0.8356	0.2273
Type II diabetes mellitus	186	620	1004	0.2372	0.008	0.0278
Total	4342	13084	18885			

1. Chi-square tests between pairwise chips were performed to test whether two chips have same percentages of SNPs in the pathways. Bonferroni correction was proceeded to correct multiple testing. P-values smaller than significant level (P<0.002) are in bold type.

### Distribution patterns of SNPs in evolutionary conserved introns and nongenic regions

**Figure 5:**
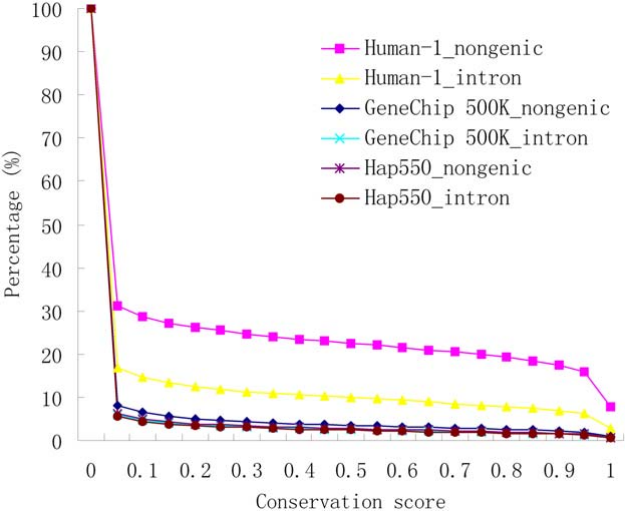
Distribution patterns of SNPs in evolutionary conserved introns and nongenic regions. The percentage of SNPs on three chips is plotted against conservation score. Human-1 contains more fractions of SNPs lying in evolutionarily conserved bases, according to its original design.

Highly evolutionarily conserved regions across species may contain unknown genes, for example, microRNA coding genes, or regulatory elements such as cis enhancers. It is important to survey the SNPs in conserved regions both outside genes and in introns. We plot SNP percentage against conservation score of sequence base in **Figure 5**. If conservation threshold is set as 0.9, about two percent SNPs of GeneChip 500K and Hap550 locate in conserved intron and nongenic bases. Due to enrichment of SNPs in conserved sequence, Human-1 contains more fractions of SNPs lying in the evolutionarily conserved bases (17.5% for nongenic regions, 6.9% for intron). When considering tagged SNPs, the difference between chips is inconspicuous (**Figure S2**) and about 3% of SNPs have scores exceed conserved threshold.

## Discussion

Various GWA studies have been performed to examine the role of common genetic variants in complex diseases and traits, taking advantage of recent advances in high-throughput SNP genotyping technologies. It has been proved that both the direct and the indirect approaches are capable of identifying disease variants in genes. For example, an intron SNP (rs7903146) and nearby SNPs in LD with it in transcription factor 7-like 2 gene (TCF7L2) gene had been replicated in several researches with different approaches [1]–[3]. In our study, we show that the current high-density SNP chips provide very high coverage in genic regions and can capture most of known common disease variants under HapMap frame, no matter which SNP selection strategy is used. Acting as a hybrid of the indirect and the direct approaches to evaluate whole-genome association, Human-1 highlights SNPs enriched in genes and evolutionarily conserved regions. Therefore, we consider it as a typical chip of direct gene-centric approach and calculate tagged SNPs in HapMap. Although the indirect approaches using quasi-random SNPs or LD-based tagSNPs focus on common variants, irrespective of their genic location, they perform as good as the direct approach in genic regions via their high density and coverage. Our evaluation of coverage and characteristics is based on the Phase II HapMap data, which is the largest catalogue of common SNPs with genotyping information till now. Since most of SNPs on three chips show concordance with HapMap SNPs, it is reasonable though not very fair to use HapMap for characterizing the chips. To simplify the procedure, we only used the pairwise aggressive algorithm and two LD thresholds (r^2^≥0.8 and r^2^≥0.5) to capture tagged SNPs in CEU. On the whole, all three chips can cover more than one half of common SNPs from HapMap in genic regions.

It is hard to estimate the ability of the approaches to capture disease variants. Then it is available to estimate whether the known susceptibility SNPs to common diseases are covered by ongoing GWA studies using chips. There are some public databases such as OMIM [15], GAD [18], HGV [19], HGMD [20] collecting variants that lead to human diseases and phenotypes variation. However, the number of susceptibility SNPs is limited in nowadays databases. Thus, we calculate and compare the ability of the chips to capture 1338 nonredundant SNPs in OMIM that affect susceptibility of human diseases, most of which are nonsynonymous mutations. With respect to common SNPs (MAF≥0.05) HapMap genotyped, the chips perform well and can capture most of them. According to rare SNPs, they are mostly ignored by the chips in current stage. It had been suggested that a genome-wide genotyping product could be coupled with a gene-centric SNP set such as SeattleSNPs Program for Genomic Application [21] to improve the ability of covering rare SNPs.

Several factors besides genomic coverage and map density can affect the power of gene-centric GWA studies. One is the proportion of SNPs in functionally important genic regions and conserved noncoding sequences. Nonsynonymous coding SNPs and SNPs in promoters are most traditionally attractive for their potential altering protein function [22], altering transcript splicing [23] destabilizing protein 3D structure and reduce protein solubility [24], and altering regulatory ability [25]. Evolutionarily conserved regions across species may contain functionally important elements, for example, cis- regulatory elements [26] and replication start points [27], or unknown genes such as microRNA coding genes [28]. Many computational approaches based on multi-species alignment have been developed to search regulatory elements in evolutionarily conserved regions [29], [30]. Follow-up experiments also validated the potential function of transcriptional regulation and development association [31], [32]. It is also important to characterize the conservation property of SNPs outside genes and in introns [33]. Our results show that the indirect approaches have highly similar patterns in these important function sequences. Although difference exhibits between the indirect and the direct approaches for SNPs on the chip, it becomes inconspicuous after considering tagged SNPs.

Another important factor is the enrichment of SNPs in ontologies and pathways. Ontologies and pathways are essential and widely used for differential expression in pathway level [34], protein-protein interaction (PPI) analysis [35] and constructing PPI network [36]. Thus, it is reliable to analyze gene-gene interaction [37] and construct genetic interaction network via SNPs-enriched ontologies and pathways [38], [39]. Lesnick et al. proposed a genomic pathway approach to construct models of axon-guidance pathway SNPs that can predict the susceptibility of Parkinson disease [39]. It hints the potential ability of mining disease associated ontologies and pathways using high-density SNP chips. We examined whether SNPs of Human-1, GeneChip 500K and Hap550 distribute in the same ontologies and pathways via genes and tested the hypothesis that the same fraction of SNPs on the chips distribute in the KEGG human disorder pathways. On most occasions, Human-1 has the similar fractions as GeneChip 500K and Hap550. Significant differences are observed in some pathways especially when considering tagged SNPs. Since Hap550 almost cover HapMap and Human-1 contains a limited subset (**Figure 1**), it implies that SNPs of Human-1 are not evenly distributing in genes and pathways.

In this year, Affymetrix (http://www.affymetrix.com) and Illumina (http://www.illumina.com) released their one million commercial SNP chips, which are most high density SNP chips available till now. We can expect that the SNP chips will cover most of the human common SNPs and density will not be a common topic in the coming future (maybe in five years). One possible future direction of developing SNP chips is population-specific chips. The current chips aim at common SNPs of three representative populations (CEU, JPT+CHB, YRI), while CEU matches the SNP sets best and YRI worst. Although YRI-specific SNP chip has been designed, the population-specific (especially the populations other than three major populations) chips are needed since various researches have shown that the portability of tagSNPs across some populations is not satisfying [40]–[43]. Another possible direction is developing rare SNPs-based chips, however, potential large sample size still obstructs this way. A most realistic direction is developing disease/pathway specific chips for specific researches. This is quite different with previous candidate pathway/gene studies. The future disease/pathway specific chips will have the advantages of both high density and research specificity. That means all possible disease-oriented SNPs in pathways/genes are included, which is based on the knowledge and Bioinformatics annotations of the diseases. This will separate the “discovering susceptible SNPs” stage by WGA chips and “replicating associations and constructing genetic models” stage by specific chips similarly as we did with resequencing and genotyping. This will reduce the cost and increase the sample size greatly. Thus, WGA studies era will be realistic. For the above reason, Bioinformatics will be deeply involved in the designing of the chips, analyzing the data and constructing the models. Our analysis in this research will be an exploration in this future field.

## Materials and Methods

### Data sets

Three genome-wide SNP chips (Human-1, GeneChip 500K and Hap550) were selected for evaluations, representing gene-centric, quasi-random SNPs and LD-based tagSNPs approaches, respectively. SNP lists were downloaded from their websites. Since most of SNPs on three chips show concordance with HapMap SNPs, we used Phase II HapMap CEU (release 21) data [14] to evaluate coverage of SNP chips in Caucasian population.

### LD-tagged SNPs and coverage calculation

All SNPs of three chips were mapped to HapMap to ensure SNPs on the chip, and these SNPs were considered as tag SNPs to capture LD-tagged SNPs. Frequency and LD data of SNPs were downloaded from HapMap website. We simply used pairwise aggressive algorithm [44] to ascertain SNPs that have pairwise r^2^ larger than the specific thresholds (r^2^≥0.8 and r^2^≥0.5). Since Human-1 acts as a hybrid of the indirect and the direct approaches, we also calculate its tagged SNPs to get the maximum coverage. When calculating coverage, only common SNPs (MAF≥0.05) were considered. Coverage is estimated by SNPs (chip+tagged) divided by all SNPs in HapMap.

### Bioinformatics annotation for SNPs

All SNPs were annotated using National Center for Biotechnology Information (NCBI) dbSNP (build 126) [45]. Each SNP from various data sets was mapped to dbSNP via ref SNP (rs). SNPs without an rs number or not presented in current dbSNP would be ignored. A SNP was first annotated in gene or nongenic regions. Then genic SNP would further be annotated with five functional classifications: flank (within 2 kb 5′ or 500 bp 3′ of a gene, originally named locus in dbSNP), utr (5′ and 3′ untranslated region), synonymous (synonymous coding SNP), nonsynonymous (nonsynonymous coding SNP) and intron (including splice-site SNPs).

OMIM (Nov, 2006) [15] deposited 1338 nonredundant SNPs that affect human diseases or phenotypes variation, most of which are nonsynonymous mutations. These SNPs were mapped to HapMap data and evaluated the capturing ability of the SNP chips. The procedure was same as coverage calculation.

We examined whether SNPs of Human-1, GeneChip 500K and Hap550 distribute in the same ontologies and pathways via genes. SNPs were mapped to GO annotations and KEGG pathways via NCBI dbSNP [45] and Entrez Gene [46]. We plotted the distribution of SNPs in categories with three concentric rings for the chips. In addition, we compared distribution difference between the paired chips in KEGG human disorders pathways. Chi-square tests between pairwise chips were performed to test whether two chips have same percentages of SNPs in the pathways. Bonferroni correction was proceeded to correct multiple testing.

Base-by-base conservation scores for human bases were downloaded from UCSC Conservation Track [47] which used phastCons [48] to calculate conservation scores across 28 mammalian species. SNPs in nongenic and intron regions were mapped to the bases to attain conservation scores. Conservation scores can be considered as probabilities that each SNP lies in a conserved element [48].

## Supporting Information

Figure S1 Percentage of SNPs in genic and nongenic regions. Shows the percentage of disease variants captured by three chips either directly (chip) or through linkage disequilibrium (r^2^≥0.8).
(1.18 MB DOC)

Figure S2 Distribution patterns of SNPs in evolutionary conserved introns and nongenic regions. The percentage of SNPs (r^2^≥0.8) on three chips is plotted against conservation score.
(1.31 MB DOC)

Table S1 MAF of 159 disease SNPs (r^2^≥0.5) in HapMap
(0.03 MB DOC)

Table S2 Distribution differences of SNPs (r^2^≥0.8) in KEGG human diseases pathways. 1. Chi-square tests between pairwise chips were performed to test whether two chips have same percentages of SNPs in the pathways. Bonferroni correction was proceeded to correct multiple testing. P-values smaller than significant level (P<0.002) are in bold type.
(0.07 MB DOC)
