# Supplementary figures and images for: Gene-Centric Characteristics of Genome-Wide Association Studies
Source: PLoS One. 2007 Dec 5;2(12):e1262. doi: 10.1371/journal.pone.0001262 (PMC2092383; doi:10.1371/journal.pone.0001262)

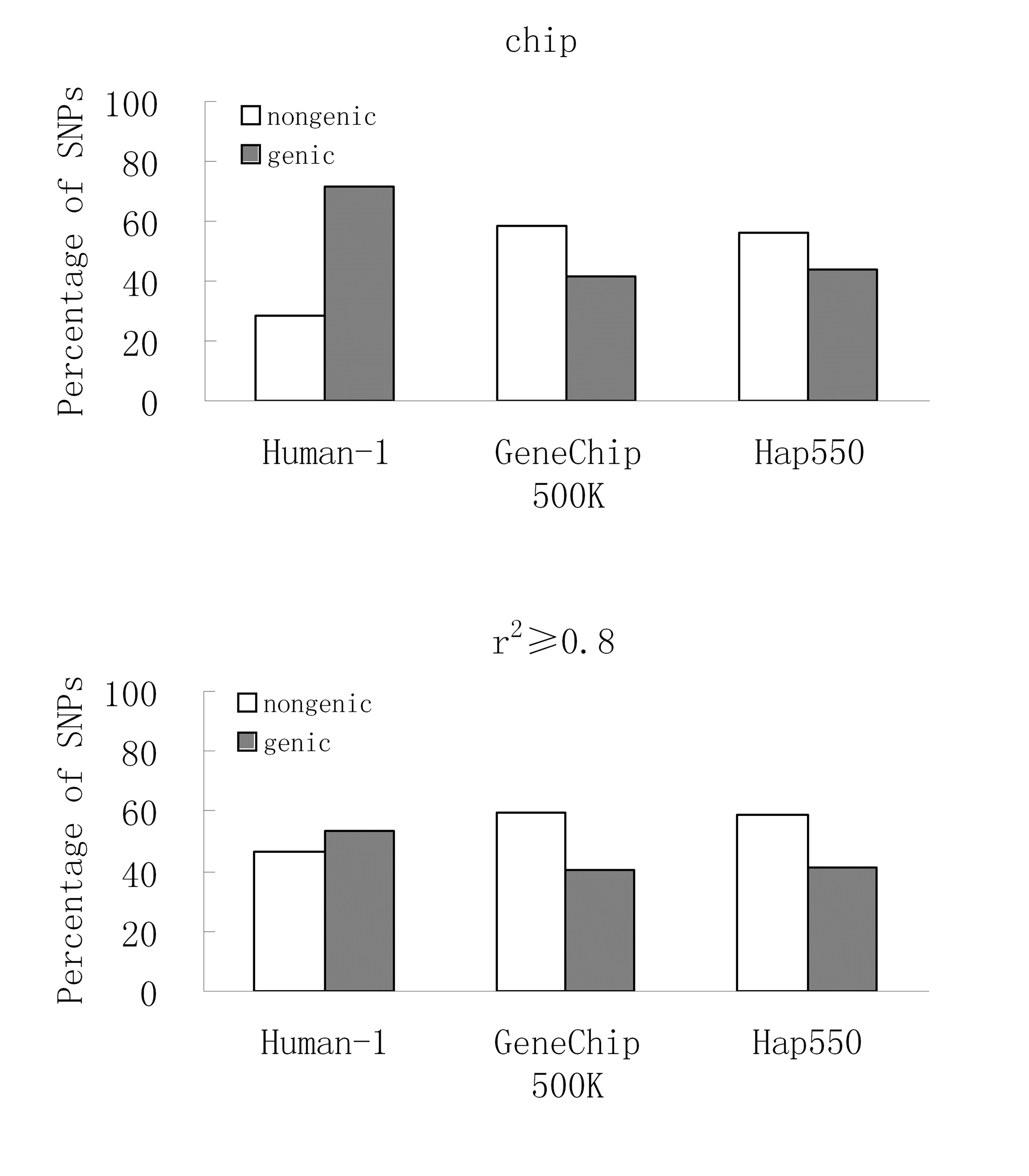

Supplement: Figure S1 — Percentage of SNPs in genic and nongenic regions. Shows the percentage of disease variants captured by three chips either directly (chip) or through linkage disequilibrium (r2≥0.8). (1.18 MB DOC) [file pone.0001262.s001.doc]

**
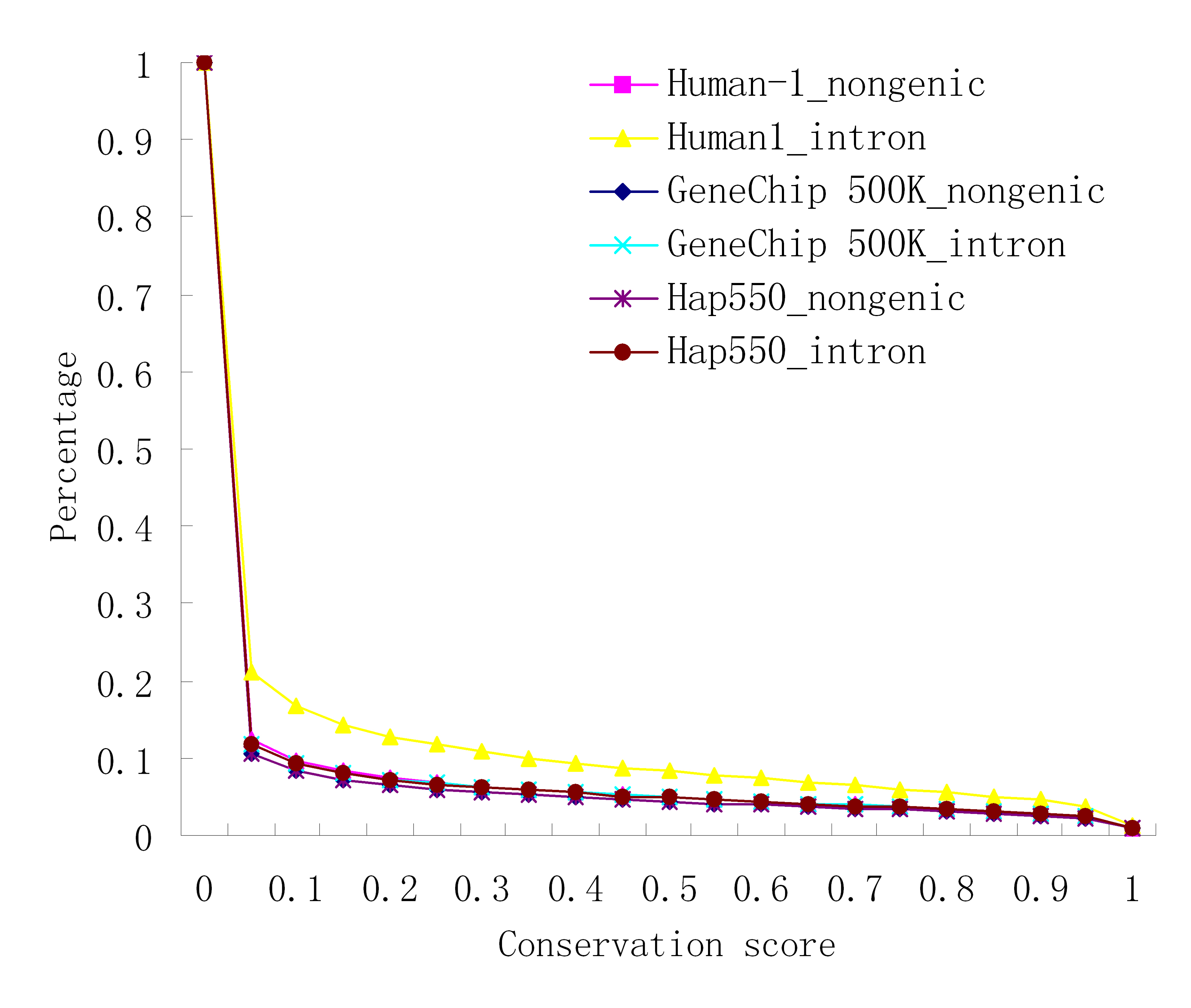
**

Supplement: Figure S2 — Distribution patterns of SNPs in evolutionary conserved introns and nongenic regions. The percentage of SNPs (r2≥0.8) on three chips is plotted against conservation score. (1.31 MB DOC) [file pone.0001262.s002.doc]
