# Supplementary material for: Gene-Centric Characteristics of Genome-Wide Association Studies
Source: PLoS One. 2007 Dec 5;2(12):e1262. doi: 10.1371/journal.pone.0001262 (PMC2092383; doi:10.1371/journal.pone.0001262)
Supplement: Table S1 — MAF of 159 disease SNPs (r2≥0.5) in HapMap (0.03 MB DOC) [file pone.0001262.s003.doc]

|  | Captured (%) | Uncaptured (%) | Total (N) |
| --- | --- | --- | --- |
| MAF≥0.05 | 94 | 6 | 100 |
| MAF<0.05 | 25 | 75 | 59 |
