# Supplementary material for: Gene-Centric Characteristics of Genome-Wide Association Studies
Source: PLoS One. 2007 Dec 5;2(12):e1262. doi: 10.1371/journal.pone.0001262 (PMC2092383; doi:10.1371/journal.pone.0001262)
Supplement: Table S2 — Distribution differences of SNPs (r2≥0.8) in KEGG human diseases pathways. 1. Chi-square tests between pairwise chips were performed to test whether two chips have same percentages of SNPs in the pathways. Bonferroni correction was proceeded to correct multiple testing. P-values smaller than significant level (P<0.002) are in bold type. (0.07 MB DOC) [file pone.0001262.s004.doc]

| Disease | Number of SNPs in pathways | | | Pairwise comparison p-value^1^ | | | |
| --- | --- | --- | --- | --- | --- | --- | --- |
|  | Human-1 | 500K | Hap550 | | Human-1:  500K | Human-1:  Hap550 | 500K:  Hap550 |
| Alzheimer's disease | 462 | 634 | 841 | | 0.0119 | 0.0542 | 0.4214 |
| Amyotrophic lateral sclerosis (ALS) | 298 | 510 | 628 | | 0.3708 | 0.6319 | 0.5966 |
| Basal cell carcinoma | 532 | 732 | 983 | | 0.008 | 0.0729 | 0.2652 |
| Cholera | 466 | 783 | 1099 | | 0.4234 | 0.0087 | 0.0359 |
| Chronic myeloid leukemia | 1468 | 1884 | 2262 | | **5.1E-10** | **4.5E-16** | 0.0742 |
| Colorectal cancer | 1885 | 2755 | 3400 | | 0.003 | **4.4E-05** | 0.2661 |
| Dentatorubropallidoluysian atrophy (DRPLA) | 755 | 1567 | 2084 | | **9.0E-09** | **1.8E-12** | 0.1853 |
| Endometrial cancer | 1753 | 3077 | 3871 | | 0.0034 | 0.007 | 0.6809 |
| Epithelial cell signaling in Helicobacter pylori infection | 1016 | 1404 | 1763 | | **0.0004** | **6.5E-05** | 0.7377 |
| Glioma | 1180 | 1852 | 2393 | | 0.5843 | 0.9018 | 0.6054 |
| Huntington's disease | 435 | 573 | 648 | | 0.0022 | **5.0E-07** | 0.0429 |
| Maturity onset diabetes of the young | 131 | 198 | 289 | | 0.604 | 0.4505 | 0.1347 |
| Melanoma | 1234 | 1968 | 2477 | | 0.8957 | 0.68 | 0.7503 |
| Neurodegenerative Disorders | 971 | 2007 | 2488 | | **1.8E-10** | **2.8E-09** | 0.4159 |
| Non-small cell lung cancer | 1177 | 2508 | 3238 | | **3.6E-15** | **6.7E-18** | 0.571 |
| Pancreatic cancer | 1342 | 1740 | 2176 | | **1.2E-08** | **1.4E-10** | 0.6205 |
| Parkinson's disease | 428 | 1086 | 1349 | | **9.6E-16** | **4.3E-15** | 0.5776 |
| Pathogenic Escherichia coli infection | 581 | 1071 | 1317 | | 0.0073 | 0.0341 | 0.4272 |
| Prion disease | 233 | 321 | 445 | | 0.08 | 0.4273 | 0.2382 |
| Prostate cancer | 1443 | 1959 | 2537 | | **3.3E-06** | **1.4E-05** | 0.5456 |
| Renal cell carcinoma | 1279 | 1852 | 2186 | | 0.0065 | **1.1E-06** | 0.0219 |
| Small cell lung cancer | 2311 | 3540 | 4620 | | 0.1068 | 0.4811 | 0.2595 |
| Thyroid Cancer | 515 | 689 | 876 | | 0.0021 | 0.0013 | 0.9964 |
| Type I diabetes mellitus | 435 | 836 | 1119 | | 0.0023 | **4.2E-05** | 0.2638 |
| Type II diabetes mellitus | 789 | 1505 | 2007 | | **0.0001** | **2.0E-07** | 0.1681 |
| Total | 23119 | 37051 | 47096 | |  |  |  |
